# Mass-like Dieulafoy’s lesion associated with advanced gastric cancer at the antrum of stomach: a case report and literature review

**DOI:** 10.1186/s13000-017-0663-y

**Published:** 2017-10-10

**Authors:** Hsi-Lan Huang, Chi Yan Leung, Chien-Jui Cheng

**Affiliations:** 1 Department of Pathology, Taipei Medical University Hospital, No.252, Wu Hsing Street, Taipei City, 110 Taiwan; 2 Department of Global Health Policy, Medical Building No. 3, Hongo Campus, University of Tokyo, 7-3-1, Hongo, Bunkyo-ku, Tokyo, Japan

**Keywords:** Advanced gastric cancer, Antrum, Dieulafoy’s lesion, Caliber-persistent artery, Mass-like, Gastrointestinal bleeding

## Abstract

**Background:**

Dieulafoy’s lesion, also known as a caliber-persistent artery, is a shallow, small, and rare lesion that occurs along the lesser curvature of proximal stomach. It is rare for a Dieulafoy’s lesion to present as a mass-like lesion that coexists with gastric cancer. To our best knowledge, we report the first case and histopathological pictures of a mass-like Dieulafoy’s lesion coexisting with advanced gastric cancer in the antrum of the stomach.

**Case presentation:**

A 57-year-old female presented with a 6-month history of intermittent epigastric dull pain and dyspepsia. Subsequent upper gastrointestinal endoscopy revealed a friable mass that was located between the distal antrum and the pyloric ring. Biopsy revealed it to be an intestinal type adenocarcinoma. Subtotal gastrectomy was performed after neoadjuvant chemotherapy. Grossly, a large irregular plaque-like tumor lesion was noted at the anterior wall of the distal antrum and pylorus ring near the lesser curvature, measuring 5.6 × 4.8 × 1.0 cm. Histopathological examination of the resected stomach revealed that the plaque-like lesion largely consisted of numerous abnormally large-caliber and tortuous arteries in the submucosa. The increased fibrosis of the submucosa resulted in the formation of elevated plaque. The intestinal type adenocarcinoma was noted to be largely confined to the mucosa layer, with focal submucosal and muscular propria involvement. The patient was discharged one week after the subtotal gastrectomy, and she was alive and well 17 months after discharge, with no major complications.

**Conclusion:**

This is the first case of a mass-like Dieulafoy’s lesion coexisting with advanced gastric cancer at the distal antrum area. This case highlights the possibility of life-threatening gastric bleeding after mucosal resection or biopsy that could be encountered by endoscopists.

## Background

Dieulafoy’s lesion (DL), a rare arterial malformation of stomach, is an important cause of gastrointestinal bleeding. Extremely rare in the antrum, 60% of DL are confined to within 6 cm from the esophago-cardiac junction of stomach. Only 18 cases of stomach DL were ever reported to coexist with gastric cancer, and only 2 of the 18 cases were advanced gastric cancers (Table 1) [1–7]. Although there is no definite pathophysiologic explanation regarding the association between DL and gastric cancer, discrete uniform pathologic descriptions have been reported in all cases - the location of DL constantly corresponded to the location of the gastric cancer [5, 8]. To the best of our knowledge, no single English report describes a mass-like DL associated with advanced gastric carcinoma in the antrum. We report herein a case and histopathological pictures of a mass-like DL coexisting with advanced gastric cancer in the antrum.

**Table 1:** Report Cases of gastric cancer found associated with Dieulafoy’s lesion

	No.	Age/ Gender	DL Location	Size (mm)	Type	Cancer location	Histology	Involved layer	Endoscopy Diagnosis	Author, Year
Early Gastric Cancer	1	66/M	Unknown	Unknown	IIc + III	On Dieulafoy	Unknown	sm	Benign	Sasaki, 1982
	2	63/M	C. Ant	20 × 20	IIc	On Dieulafoy	tub	sm	Benign	Maeba, 1986
	3	47/F	M. Less	Unknown	IIc	On Dieulafoy	tub2	m	Benign	Fujimori, 1988
	4	42/M	C. Ant	Unknown	IIc	On Dieulafoy	tub2	sm	Benign	Fujimori, 1988
	5	32/F	C. Ant	16 × 12	IIc + III	On Dieulafoy	tub2	m	IIc	Kawamura, 1991
	6	65/M	C. Post	22 × 17	IIc	On Dieulafoy	tub1	sm	IIc	Natsugoe, 1991
	7	41/M	C. Post	15	IIc	On Dieulafoy	sig	m	Benign	Leone, 1995 [1]
	8	70/M	C. Ant	70 × 50	IIc	On Dieulafoy	tub1	sm	Benign	Yasutomo, 1995
	9	34/F	C. Ant	25 × 25	IIc + III	On Dieulafoy	sig	sm	IIc + III	Fuke, 1996
	10	71/M	C. Less	30 × 20	IIc	On Dieulafoy	tub2	m	Benign	Hisa, 1997
	11	56/M	M. Post	20 × 20	IIc	On Dieulafoy	tub1	sm	Benign	Wakahara, 2000 [2]
	12	42/F	C. Less	40 × 20	IIc	On Dieulafoy	tub2	sm	IIc	Shimomatsuya, 2000
	13	45/M	M. Ant	10	IIc	On Dieulafoy	sig	m	IIc	Ikeda, 2001
	14	48/M	C. Post	Unknown	IIc	On Dieulafoy	sig	sm	Benign	Kishikawa, 2003 [3]
	15	69/M	C. Post	50 × 40	IIa	On Dieulafoy	tub1	sm	Benign	Taketsuka, 2006 [4]
	16	75/M	Stump	4 × 3	IIc	On Dieulafoy	tub1	sm	0-IIc	Gurzu, 2013 [5]
Advanced Gastric Cancer	1	48/M	M.Ant	20 × 20	IIc	On Dieulafoy	sig	mp	Benign	Taniguchi, 1992
	2	56/M	C. Less	50 × 25	IIc + IIa	On Dieulafoy	tub2	mp	Benign	Kishimoto, 2005 [6]
	3†	57/F	A. Ant	56 × 48	III	On Dieulafoy	tub2	mp	III	Present Case, 2016

C, upper third; M, middle third; A. Lower third; Ant, anterior wall; Post, posterior wall; Less, lesser curvature; Ca, cancer; tub, tubular adenocarcinoma; tub 1, well-differentiated adenocarcinoma; tub 2, moderately differentiated adenocarcinoma; sig, signet–ring cell carcinoma; m, mucosa; sm, submucosa; mp, proper muscle; † This case

## Case presentation

**Fig. 1:**
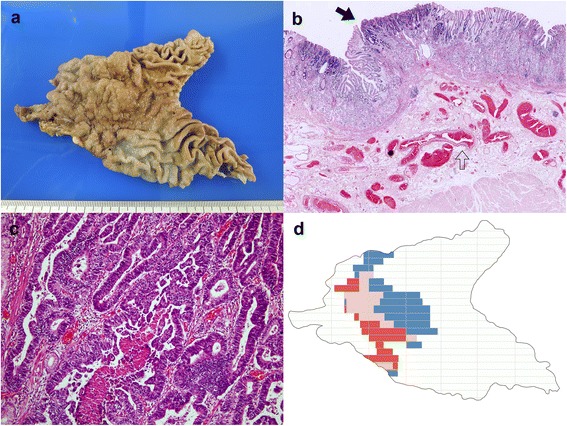
**a** Gross features of the subtotal gastrectomy specimen. A protruded tumor was identified at the anterior wall of the distal antrum and pylorus ring near the lesser curvature. **b** Numerous abnormally large-caliber tortuous arteries in submucosa with increased submucosal fibrosis, resulting in formation of an elevated plaque. **c** The well differentiated adenocarcinoma was mostly noted within the mucosa (*arrow*). Beneath the adenocarcinoma, abnormally large-caliber tortuous arteries were identified in the submucosa with increased submucosal fibrosis (*hollow arrow*). **d** A schematic drawing of the resected specimen showed the distribution of the gastric cancer and Dieulafoy’s lesions. The *blue* areas represented Dieulafoy’s lesions without overlying gastric cancers. The *red* areas showed the gastric cancer without underlying Dieulafoy’s lesion. The *pink* areas represented coexistence of gastric cancer and Dieulafoy’s lesions (gastric cancer observed just above the Dieulafoy’s lesions)

**Fig. 2:**
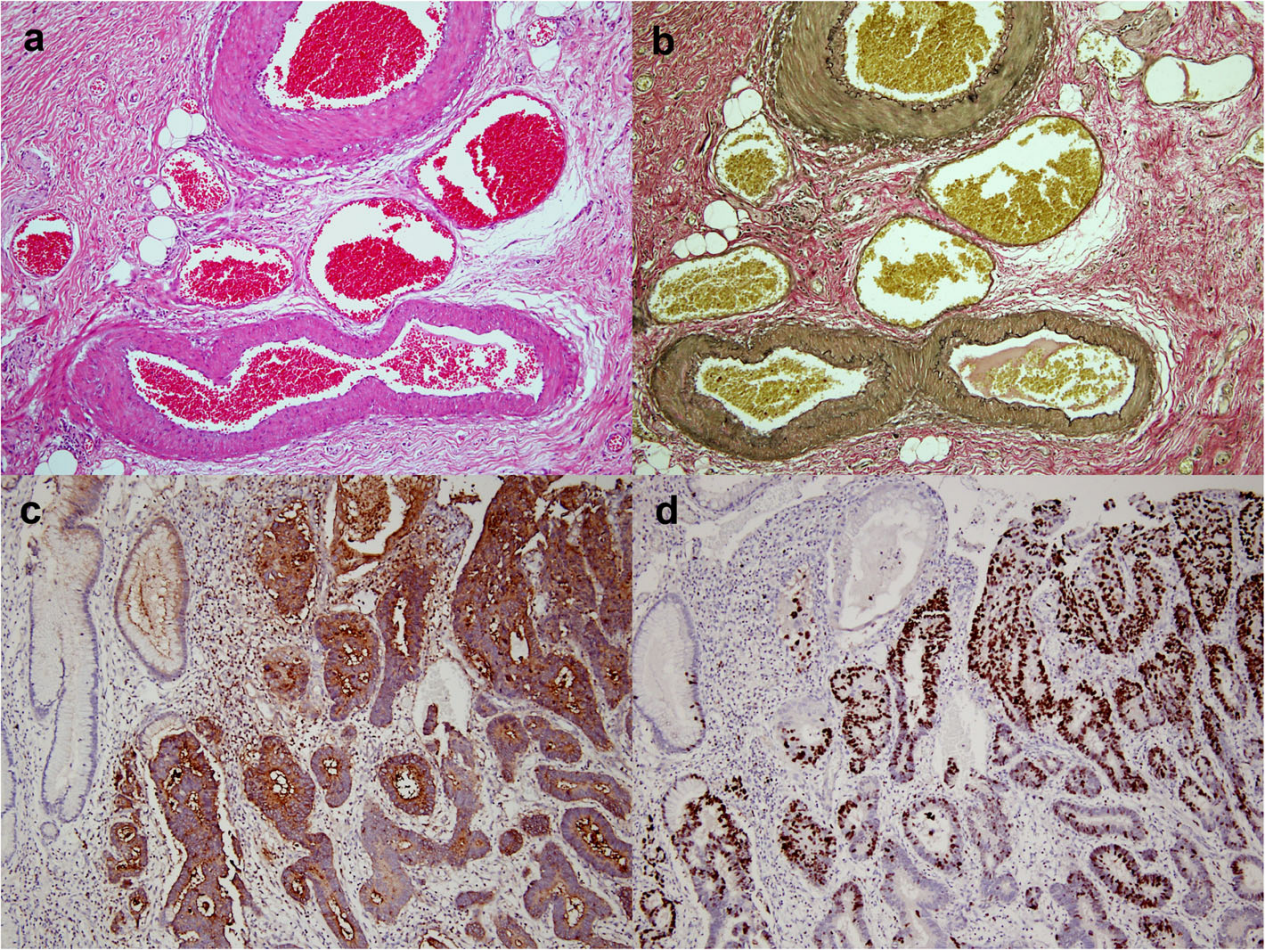
**a** Histopathological findings of gastric caliber persistence or Dieulafoy’s lesion. Oversized vascular submucosal clusters containing tortuous vessels could be seen within the submucosal layer. **b** Elastic stain demonstrated the persistent caliber arteries. At the cancerous area, immunohistochemistry studies revealed diffusely positive for CEA (**c**) and an increased proliferative index under Ki-67 study (**d**)

A 57-year-old female presented with a 6-month history of intermittent epigastric dull pain and dyspepsia. No gastrointestinal hemorrhage, use of non-steroidal anti-inflammatory drugs, excessive alcohol ingestion, or chronic liver disease was reported. A positive fecal occult blood test was reported 3 months prior to admission. Hemoglobin and hematocrit were 10.1 g/dL and 28%, respectively, with an otherwise normal full blood picture. Subsequent upper gastrointestinal endoscopy revealed a friable mass which extended from the distal antrum to the pyloric ring. Routine biopsy showed the presence of intestinal type adenocarcinoma. Abdominal computerized tomography showed a tumor mass originating from the anterior wall of the antrum with a suspicion of lymph node metastasis. Subtotal gastrectomy was performed after neoadjuvant chemotherapy. Grossly, a large irregular plaque-like lesion, sized 5.6 × 4.8 × 1.0 cm, was noted at the anterior wall of the distal antrum and pylorus ring near the lesser curvature (Fig. 1a). Postoperatively, the diagnosis of DL was made via histopathologic examination. At microscopy, numerous abnormally large-caliber tortuous arteries were identified in the submucosa with increased submucosal fibrosis, resulting in formation of an elevated plaque (Fig. 1b). The intestinal type adenocarcinoma was mostly confined to the mucosa (Fig. 1c), with focal submucosal and muscular propria involvement. Results of the serial section performed in the cancer and non-tumor areas of the specimen are shown in Fig. 1d. The intima of the arteriolar wall exhibited focal thickening with ordinary elastic lamina (Fig. 2a, b). Two regional metastatic lymph nodes were reportedly found in the specimen. No lymphovascular invasion was identified. No *Helicobacter pylori* were seen in the regional non-neoplastic gastric mucosa. The regional non-neoplastic gastric mucosa revealed chronic gastritis with increased lymphocytes and plasma cells infiltrations. Focal intestinal metaplasia was noted. At the cancerous area, immunohistochemistry studies revealed diffusely positive for CEA (Fig. 2c) and an increased proliferative index in Ki-67 study (Fig. 2d). The patient was discharged 1 week later, and she was alive 17 months following surgery with regular follow-up.

## Discussion

DL was first reported by Gallard in 1896 and was subsequently described more accurately by Georges Dieulafoy as “exulceratio simplex” in 1898 [9]. In modern times, DL has been interpreted as a congenital anomaly characterized by abnormally dilated submucosal arteries without aneurysm formation or intrinsic mural abnormality. Unlike normal arterial growth, arteries of DL remain persistently large in diameter even in distal branches. These distinctive histopathological patterns of DLs were described by Leone et al. as a “mixture of arterial and venous anomalies” [1]. The current case showed increased and proliferative clusters of vessels with arterial morphology in the submucosa beneath the tumor area, which is consistent with the histopathological picture of DL. Most DL (60%) occur in the proximal stomach, 6 cm distal to the esophago-cardiac junction on the lesser curvature, and are supplied by the left gastric artery arising from the celiac trunk [7]. Twelve percent of DL are located at the distal antrum and are fed by the right gastric artery or an abnormal vascular anastomosis among the left and right gastric artery along the lesser curvature [8, 10]. In our case, the lesion was located at the anterior wall of the distal antrum and pylorus ring near the lesser curvature. This atypical location could pose a diagnostic challenge to endoscopists. Awareness of possible adverse events during biopsy and mucosal resection that could be caused by the rare phenomenon of DL could be easily overlooked.

Upper gastrointestinal endoscopy is the major diagnostic modality for DL, and it is effective in up to 70% of patients. DL usually presents as small pigmented shallow protrusive lesions with mild erosion and no ulceration at endoscopy. Active spurting or oozing from a mucosal defect < 5 mm in diameter is often noted. The size of DL is about 10-15 mm in width and 5-10 mm in height. Angiography can be helpful in localizing the lesion, especially in the case of acute bleeding [8]. Mass-like and papillae DL lesion in the stomach and small intestine have only been reported in two cases, and they were clinically and radiologically diagnosed as submucosal tumors instead [11, 12]. In our case, at endoscopy, the tumor appeared to be a plaque-like mass lesion with shallow ulceration and mucosal change similar to a typical gastric carcinoma, however the consistency of the tumor was soft and friable. The differential diagnosis at endoscopy included advanced gastric carcinoma and gastric lymphoma. This hybrid endoscopic finding suggests a diagnosis of mass-like DL.

The association between DL and gastric cancer is not fully elucidated. Taketsuka et al. hypothesized that repeated erosions and ulcers in the mucosa could be induced by circulation disturbances in vessels of DL, and active regeneration and dysplasia may further promote the process of carcinogenesis. This hypothesis is in line with the phenomenon that DL was located underneath the cancerous lesion in all reported cases [4]. Nonetheless, the prognosis of patients with gastric cancer concomitant with DL mainly depends on the pathologic stage of the gastric cancer.

Differential diagnosis of this case included gastric antral vascular ectasia (GAVE) and portal hypertensive gastropathy. GAVE accounts for up to 4% of all non-variceal upper gastrointestinal bleeding and is often associated with atrophic gastritis and pernicious anemia, which are known risk factors for gastric malignancy [13]. However, any association of GAVE and gastric cancer is reportedly rare [14]. Patients diagnosed are predominantly elderly females with mean age of 73 years, and they are usually associated with underlying chronic diseases, particularly cirrhosis (30%) and autoimmune diseases (62%) [15]. The characteristic endoscopic finding is “watermelon stomach” – alternating hyperemic streaks with normal mucosa radiating in a spoke-like fashion from the pylorus to the antrum. Characteristic histological pictures of GAVE include dilated capillaries and fibromuscular hyperplasia of the lamina propria, intravascular fibrin thrombi, and an increase in the mean cross-sectional area of the lumen in mucosal vessels, which were not observed in our case. Another differential diagnosis is portal hypertensive gastropathy, typically more prominent in the fundus or corpus and it is associated with liver cirrhosis.

This case highlights the possibility of mass-like DL coexisting with advanced gastric adenocarcinoma in the distal antrum. Also, our case alerts endoscopists to the risk of severe bleeding due to this unusual presentation, in view of recent preferences for the use of endoscopic modalities over surgical resection for DL treatment.

## Conclusion

In summary, the association of mass-like DL and advanced gastric carcinoma at the distal antrum is an exceptionally rare condition. The postoperative diagnosis was due to the inability to distinguish these synchronous lesions during pre-operative gastroscopy. Hence, with the unique presentation and vascular architectures, this case highlights the possibility of life-threatening massive bleeding after biopsy and mucosal resection that could be encountered by endoscopists.

## Abbreviations

DL: Dieulafoy’s lesion
GAVE: Gastric antral vascular ectasia
